# The impact of perioperative aspirin utilization on postoperative hemorrhagic complications in idiopathic normal pressure hydrocephalus: a single-center retrospective analysis

**DOI:** 10.1007/s10143-025-03459-4

**Published:** 2025-03-17

**Authors:** Ahmet İlkay İsikay, Efecan Cekic, Amin Charehsaz, Zeynep Arzum Uyaniker, Gul Yalcin Cakmakli, Rahsan Gocmen, Sahin Hanalioglu, Bulent Elibol

**Affiliations:** 1 Department of Neurosurgery, Hacettepe University, Ankara, Turkey; 2 Department of Neurology, Hacettepe University, Ankara, Türkiye; 3 Department of Radiology, Hacettepe University, Ankara, Türkiye

**Keywords:** Idiopathic normal pressure hydrocephalus (iNPH), Ventriculoperitoneal shunt, Perioperative aspirin use, Postoperative hemorrhagic complications, Neurosurgery, Antiplatelet therapy

## Abstract

**Background and Objectives:**

Idiopathic normal pressure hydrocephalus (iNPH) primarily affects older patients. Ventriculoperitoneal (VP) shunt surgery is a standard treatment. Many iNPH patients have high cardiovascular risks and require aspirin (ASA) therapy to prevent thromboembolic events. Discontinuing ASA increases the risk of these events. This study evaluates the impact of perioperative ASA use on hemorrhagic complications in iNPH patients undergoing VP shunt surgery.

**Methods:**

This retrospective cohort study included patients who underwent VP shunt surgery for iNPH from January 2020 to September 2024. Patients were divided into two groups based on perioperative ASA use: no ASA (*n* = 50) and ASA continued (*n* = 51). Data collected included demographics, surgery details, ASA dosage, and indications for ASA use. Primary outcomes were early and late postoperative hemorrhage incidences. Postoperative follow-up included MRI or CT scans at regular intervals (mean ≈ one year). Statistical analyses were performed using SPSS version 23.0, with Chi-square tests and independent samples t-tests or Mann–Whitney U tests used to analyze differences between groups.

**Results:**

The study cohort had 101 patients with a mean age of 69.5 ± 7.6 years, 41.6% female and 58.4% male. Early postoperative hemorrhage occurred in 5% of patients, including epidural (1), intraparenchymal(3), and intraventricular hematoma(1). Late postoperative hemorrhages occurred in 4% of patients ( 4 patients in the no-ASA group), with two cases each of unilateral and bilateral subdural hematoma. No significant differences in hemorrhagic outcomes were observed between the ASA continuation and non-use groups (*p* = 0.092). The mean follow-up period was 300 days. One patient died in non-ASA group due to neurodegenerative disease.

**Conclusion:**

Perioperative ASA use does not significantly impact the incidence of postoperative hemorrhages in iNPH patients undergoing VP shunt surgery. These findings suggest that ASA can be safely continued without increasing hemorrhagic risks. This is a particularly significant issue for patients with high cardiovascular risk.

## Introduction

Idiopathic normal pressure hydrocephalus (iNPH) is a reversible neurological condition characterized by gait disturbance, urinary incontinence, and cognitive impairment, primarily affecting older adults. Its symptoms often overlap with those of neurodegenerative disorders, leading to frequent misdiagnosis [1]. (Hakim-Adams triad—classically described by Salomon Hakim and R. D. Adams in 1965) [2, 3]. The pathophysiology of iNPH involves complex cerebrospinal fluid (CSF) dynamics, where impaired CSF absorption leads to ventricular enlargement without a corresponding increase in CSF pressure. The primary diagnosis still relies on the lumbar tap test, while treatment predominantly involves the ventriculoperitoneal (VP) shunt systems implantations, and risk analysis for surgery decision is essential [4]. Additionally, a smaller subset of patients undergoes endoscopic third ventriculostomy (ETV) procedures [5].

With aging, the prevalence of diseases requiring antiplatelet therapy, such as cardiovascular diseases, increases. Consequently, many iNPH patients undergoing VP shunt surgery are on anticoagulant therapy, primarily aspirin (acetylsalicylic acid, ASA). Current clinical practices often recommend stopping ASA about 5–7 days before elective surgeries to reduce the risk of bleeding. However, this approach is not without risks, particularly thromboembolic events in patients with high cardiovascular risks [6]. Several studies have highlighted the significant thromboembolic risks, such as myocardial infarction and stroke, associated with discontinuing aspirin therapy in high-risk patients.

Recent studies have shown conflicted results regarding the continuation of ASA in neurosurgery. One study demonstrated that aspirin increases the risk of subdural hematoma postoperatively in VP shunt patients in iNBH [7]. Conversely, another study found no significant differences between continuing and discontinuing aspirin in patients undergoing elective craniotomy for brain tumors [8]. Given the lack of consensus and robust data to universally support either perioperative use or discontinuation of anticoagulants, the potential for adverse cardiovascular outcomes raises essential questions about the necessity of stopping ASA.

Therefore, the continuation or discontinuation of ASA in the perioperative period remains debatable in iNPH. While its use is essential for preventing major cardiovascular events, it is also considered to increase the risk of postoperative hemorrhagic complications. The literature needs decisive studies that determine the impact of ASA on postoperative hemorrhagic complications in iNPH patients undergoing VP shunt surgery. This study aims to bridge this gap by providing empirical evidence on the safety and outcomes of ASA continuation versus discontinuation, potentially influencing clinical decisions and revising perioperative management protocols. Our findings are intended to contribute to a nuanced understanding of managing perioperative antiplatelet therapy, offering evidence that may inform safer clinical practices and influence existing guidelines on ASA management in neurosurgical procedures.

## Methods

This study was approved by the Local Institutional Review Board (Approval Decision Number: 2023/01–30, Research Number: GO 22/1064, Date: January 24, 2023).

**Fig. 1:**
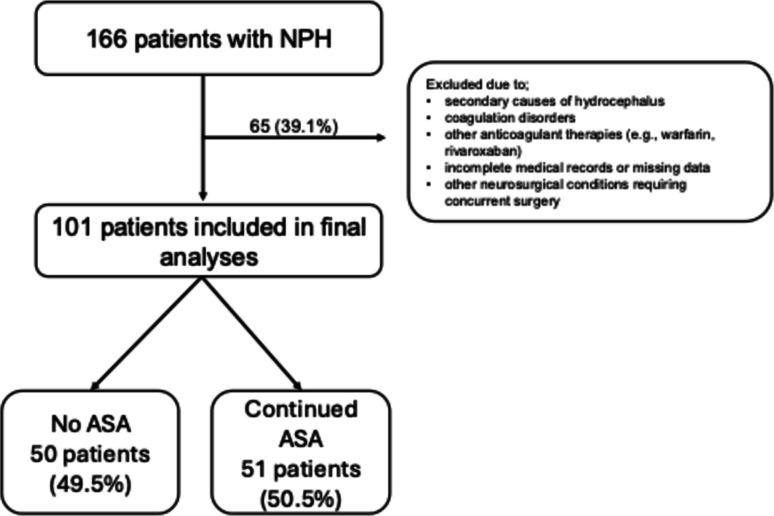
Classification of patients based on perioperative aspirin use: No ASA (n=50) vs. ASA Continued (n=51)

This retrospective cohort study was conducted at a single center to evaluate the impact of ASA use on hemorrhagic complications in patients undergoing VP shunt surgery for iNPH. The study cohort included 101 patients operated from January 2020 to September 2024. Patients were stratified into two groups based on their perioperative ASA use: no ASA (*n* = 50) and ASA continued (*n* = 51), as shown in Fig. 1. Patients in the ASA group were preoperatively evaluated by cardiology, and it was determined that discontinuing ASA posed significant cardiovascular risks. Based on this recommendation, these patients underwent surgery while continuing ASA therapy. Postoperative follow-up included regular monitoring at the 2nd week, 1st month, 3rd month, and 6th month with MRI or CT scans as needed. If shunt adjustments were necessary, patients were followed up weekly with imaging until stability was confirmed. The mean follow-up period was almost one year.

Data on demographics, surgery, ASA dosage, and indications for ASA use were collected. ASA dosages were categorized as 80 mg, 81 mg, 100 mg, and 300 mg. Surgical interventions just included adjustable VP shunt placement. The primary outcomes assessed were early and late postoperative hemorrhage incidences, categorized by type (extra-axial hematoma, intracranial hemorrhage, and intraventricular hemorrhage for early hemorrhages; unilateral and bilateral subdural hematoma for late hemorrhages).

Inclusion criteria for the study were patients diagnosed with iNPH based on clinical and radiological criteria, aged 60 years and older, who underwent VP shunt surgery and had documented use of ASA in the perioperative period. Exclusion criteria included patients with secondary causes of hydrocephalus, coagulation disorders, or those on other anticoagulant therapies (e.g., warfarin, rivaroxaban), as well as patients with incomplete medical records or missing data regarding ASA use, and those with other significant neurosurgical conditions requiring concurrent surgery. Incomplete medical records were defined as those lacking sufficient information on aspirin usage or postoperative complications, and data were collected through a combination of electronic medical records and manual chart reviews.

Statistical analyses were performed using SPSS version 23.0. The GraphPad Prism version 10.0.0 (GraphPad Software, Boston, Massachusetts, USA) was utilized to create the bar chart. Continuous variables were expressed as mean ± standard deviation (SD), and categorical data were presented as frequencies and percentages.. The differences between groups were analyzed using Chi-square tests for categorical variables and independent samples t-test or the Mann–Whitney U test for continuous variables. A p-value of less than 0.05 was considered statistically significant.

## Results

The study population comprised 101 patients with a mean age of 69.5 ± 7.6 years. The distribution by sex was 41.6% female and 58.4% male. The mean duration for hospitalization for the patients was 2.5 ± 0.8 days. All demographic characteristics are presented in Table 1.

**Table 1:** Baseline characteristics of study cohort

	All	ASA		*P* value
		None	Continued	
No. of patients	101	50 (49.5%)	51 (50.5%)	
Mean age ± SD, yrs	69.5 ± 7.6	67.7 ± 9.3	71.4 ± 5.0	**0.016**
Females	42 (41.6%)	26 (52%)	16 (31.4%)	**0.035**
Males	59 (58.4%)	24 (48%)	35 (68.6%)	
Length of hospital stay, days	2.5 ± 0.8	2.6 ± 0.8	2.4 ± 0.7	0.244
ASA doses				-
80 mg	1 (1%)		1 (2%)	
81 mg	1 (1%)	-	1 (2%)	
100 mg	47 (46.5%)		47 (92.2%)	
300 mg	2 (2%)		2 (3.9%)	
ASA Indications				-
Atrial Fibrillation	2		2	
Aortic Aneurysm	1		1	
Bypass Surgery	6	-	6	
Coronary Artery Disease	37		37	
Carotid Artery Stenting	2		2	
Pulmonary Embolism	1		1	
Post-op Thromboembolism	0	0	0	-
Post-op Hemorrhage (early)	5 (5%)	3 (6%)	2 (3.9%)	0.678
Post-op Hemorrhage (late)	4 (4%)	4 (8%)	0 (0%)	0.056
Post-op Hemorrhage (total)	9 (8.9%)	7 (14%)	2 (3.9%)	0.092
Shunt Revision	6 (5.9%)	2 (4%)	4 (7.8%)	0.678
Follow-up, days	300 [212–390]	290 [210–410]	312 [223–390]	0.726
Death	1 (1%)	1 (2%)	0 (0.0%)	0.495

Regarding ASA management, 50.5% of patients continued ASA, and 49.5% were not on ASA. The main indications for ASA use included coronary artery disease (37 patients), bypass surgery (6 patients), and atrial fibrillation (2 patients). No patients were on warfarin or rivaroxaban.

Early postoperative hemorrhage occurred in 5% of the cohort, including one case of epidural hematoma, three instances of parenchymal hemorrhage, and one case of intraventricular hemorrhage. Late postoperative hemorrhages occurred in 4% of patients, comprising two cases each of unilateral and bilateral subdural hematoma, as depicted in Table 1. Thromboembolic complications were observed in none of the patients, and all complications are detailed in Fig. 2. The rate for patients requiring shunt revision is 5.9%. Among the patients who required shunt revision, one case was due to skin dehiscence, while the others were due to infections.

No significant differences were found in the incidence of early or late hemorrhages based on ASA status (*p* = 0.092). The comparison of ASA dosage among the continued group showed no significant differences in hemorrhagic outcomes.

The follow-up period for the patients was 300 [212—390] days, and only one patient died; this patient was in the group not using ASA and passed away due to a neurodegenerative disease.

## Discussion

Our study results indicate that the continuation of ASA or its non-use does not significantly impact the incidence of postoperative hemorrhages in patients undergoing VP shunt surgery for iNPH. The ASA continuation group in our study reflects patients with higher cardiovascular risk, as these individuals underwent cardiology consultations and were advised to continue ASA to minimize thromboembolic risks. Our findings focus on evaluating the safety of perioperative ASA continuation in shunt surgery, irrespective of the indication for its use. Our data showed no significant differences in early or late hemorrhagic events among patients who continued ASA or were not on ASA. These findings suggest that the perioperative use of ASA can be safely continued in this patient population without increasing the risk of hemorrhagic complications. Our study's finding is critical as it challenges the current clinical practice of routinely discontinuing ASA before elective surgeries due to bleeding concerns. The absence of increased hemorrhagic complications in patients who continued ASA highlights the potential for revising perioperative management protocols to avoid unnecessary cessation of ASA in iNBH surgeries, which could mitigate the risk of thromboembolic events in patients with high cardiovascular risks. Our study provides empirical evidence supporting the safe continuation of ASA in iNPH patients undergoing VP shunt surgery. These findings inform clinical decision-making and potentially lead to changes in existing guidelines, promoting safer perioperative management practices for patients on long-term antiplatelet therapy.

Our findings challenge the conventional practice of discontinuing ASA before elective surgeries due to bleeding concerns. Our data suggest that maintaining ASA therapy during the perioperative period does not significantly increase the risk of postoperative hemorrhagic events. This is particularly relevant for iNPH patients with substantial cardiovascular risk profiles. The absence of significant hemorrhagic complications in our ASA-continued group aligns with the results of other studies, which found no significant differences in hemorrhagic outcomes between ASA users and non-users undergoing neurosurgical procedures, whether elective or emergent surgeries, such as brain tumors, traumatic brain injuries, and aneurysms [9–12]. Hanalioglu et al. (2020) reported hemorrhagic complication rates of 0.6% in the no-ASA group, 0.9% in the stopped-ASA group, and 0.8% in the continued-ASA group in the elective brain tumor surgeries, demonstrating that perioperative ASA use did not significantly elevate hemorrhagic risks. These findings support that ASA can be safely continued in high-risk cardiovascular patients undergoing surgery without increasing the postoperative hemorrhage [8].

Additionally, a study by Rashidi et al. (2021) on patients undergoing cerebral aneurysm surgery further supports these findings. They found that the incidence of postoperative hemorrhage was 3.1% in the ASA impact group and 3.0% in the no-ASA impact group, a difference that was not statistically significant (OR = 1.0516 [0.1187; 9.3132], *p* = 1.000). However, it is noteworthy that cardiopulmonary complications were significantly more frequent in the stopped ASA group (*p* = 0.030). This underscores the importance of balancing hemorrhagic risks with cardiovascular benefits, suggesting that ASA can be relatively safely continued in patients with increased cardiovascular risk, even in neurosurgical contexts, provided that meticulous perioperative management is maintained [13].

The potential thromboembolic risks associated with ASA discontinuation are a significant concern for patients with high cardiovascular risk. Although our study focused on hemorrhagic outcomes, it is crucial to balance these findings with the cardiovascular risks of stopping ASA therapy. Discontinuation of ASA can lead to increased incidences of myocardial infarction and stroke, as supported by various cardiovascular studies [14–16]. Therefore, our results advocate for a nuanced approach where the continuation of ASA is considered in patients with high cardiovascular risk, provided the bleeding risks remain manageable. A recent study by Rychen et al. (2023) investigated the risks and benefits of a new perioperative management protocol of continuation or ultra-early resumption of antithrombotics (AT) in elective cranial procedures. Their findings revealed that the rate of hemorrhagic complications was 4% in the AT group, 6% in the control group, and 7% in the historical AT group (*p* = 0.5). The rate of thromboembolic complications was 5% in the AT group, 8% in the control group, and 7% in the non-AT group (*p* = 0.7) [17]. These results indicate that the continuation or ultra-early resumption of AT does not increase the risk of hemorrhagic complications and may protect against thromboembolic events. This study further supports the safety of maintaining ASA during the perioperative period in neurosurgical patients, emphasizing the potential benefits of reducing thromboembolic risks without significantly increasing hemorrhagic complications. Although ventriculoperitoneal shunt surgery is not a lengthy procedure, the higher average age of iNPH patients warrants careful consideration of thromboembolic risks. The careful perioperative management of anticoagulant therapy in patients with iNPH is essential to balance the risks of thromboembolic events and hemorrhagic complications. Although our study did not directly evaluate anticoagulant therapy, previous studies have demonstrated the importance of individualized management to minimize these risks.

Birkeland et al. reported a significant association between ASA use and increased risk of SDH in NPH patients following shunt implantation, with a hazard ratio of 12.8 (95% CI 3.1–53) [7, 18]. Similarly, the study by Gasslander et al. (2021) identified male sex, antiplatelet medication use, and lower opening pressure at surgery as significant risk factors for SDH in iNPH patients [19]. While these studies highlight a potential association between ASA use and SDH risk, our findings did not demonstrate a significant difference in the incidence of SDH or other hemorrhagic complications between patients who continued ASA and those who did not use ASA. Furthermore, no significant differences were observed in early or late hemorrhagic events between the two groups. This discrepancy may stem from differences in study design, patient populations, perioperative management protocols, and follow-up durations. Specifically, the longer follow-up periods in some studies may have captured delayed complications that were not observed in our cohort, which had an average follow-up of approximately 300 days.In our study, patients underwent regular postoperative monitoring, and shunt settings were adjusted as needed to prevent subdural effusion. This meticulous management approach likely contributed to mitigating the risk of SDH and other complications. These findings underscore the importance of individualized perioperative care and highlight that, under appropriate monitoring and management, ASA continuation does not appear to substantially increase the risk of SDH in iNPH patients undergoing shunt surgery.

In another study, Hamouda et al. (2024) performed a retrospective analysis of 234 patients with iNPH. They found that patients receiving systemic anticoagulation had a higher incidence of tract hemorrhage than those not (11.1% vs. 2.5%; *P* = 0.03). However, the overall rates of hemorrhagic complications were similar between the two groups [20]. This study reinforces our observation that the use of anticoagulants, including aspirin, does not substantially elevate the overall risk of hemorrhagic complications after shunt surgery. Within our group, 2.6% of patients experienced early postoperative bleeding, whereas 5.2% experienced late postoperative hemorrhage. There were no notable variations in occurrence based on ASA status. Significantly, our series did not include any instances of tract bleeding. Therefore, as suggested by previous studies, the careful perioperative management of anticoagulant or antiplatelet therapy in patients with iNPH is essential to balance the risk of thromboembolic events against the potential for hemorrhagic complications, ensuring optimal surgical outcomes.

The European multicenter study by Feletti et al. (2018) comprehensively analyzes VP shunt complications in iNPH patients. The study reported that 26% of patients experienced symptomatic under-drainage, 9% experienced symptomatic over-drainage, and 37% required shunt adjustments. Malposition was identified in 7% of cases, infections in 0.9%, subdural hematomas in 6%, and hygromas in 9%. These complication rates are within the expected range for VP shunt procedures and highlight the importance of meticulous perioperative and postoperative management to minimize these risks [21].

Our study findings are consistent with the results reported by Sundström et al. (2018), who identified SDH as a common complication in patients with iNPH undergoing VP shunt surgery, with an incidence rate of 10%. However, contrary to their findings, where the use of adjustable shunts was shown to mitigate the severity of SDH by allowing non-invasive pressure adjustments [22], our data indicate that the incidence of SDH did not significantly differ among patients who continued or discontinued ASA use perioperatively. This suggests that, in our cohort, the continuation or discontinuation of ASA does not substantially influence the risk of developing SDH postoperatively.

Our study demonstrates that the continuation of aspirin (ASA) during ventriculoperitoneal (VP) shunt surgery in iNPH patients does not significantly increase the risk of hemorrhagic complications. However, the findings by Gao et al.(2024) indicate that long-term ASA therapy significantly elevates hemorrhagic risks during extraventricular drainage (EVD) procedures. The discrepancy may be attributed to fundamental differences between these two neurosurgical interventions. EVD involves the placement of a catheter into the ventricles, often in emergency settings with increased vascular fragility and coagulopathy, which may heighten the risk of bleeding. In contrast, VP shunt surgery is typically an elective procedure with a controlled surgical environment, allowing for meticulous hemostasis and careful perioperative management. These distinctions underscore the importance of considering the specific surgical context and patient characteristics when evaluating the safety of ASA use [23].

### Limitations and future directions

Our study has several limitations. Conducting the study in a single-surgeon setting may introduce potential biases related to surgical technique and decision-making, and while consistent perioperative procedures reduce variability, they may limit the generalizability of our findings to other surgical settings. Additionally, the relatively small sample size limits the statistical power to detect rare complications, such as postoperative hemorrhage. Furthermore, while we evaluated the safety of ASA continuation based on hemorrhagic risks, we did not directly assess thromboembolic outcomes. Patients in the ASA group were preoperatively evaluated by cardiology and deemed at high thromboembolic risk, which guided the decision to continue ASA therapy.

Despite these limitations, our study provides valuable insights into the safety of perioperative ASA use, reflecting real-world clinical decision-making. Future multicenter studies with larger cohorts and extended follow-up periods are essential to validate these findings and assess both the hemorrhagic and thromboembolic outcomes of ASA continuation. Additionally, emerging technologies such as artificial intelligence and 3D modeling offer promising opportunities for enhancing surgical planning and optimizing patient-specific management, ultimately improving outcomes and reducing complication rates [24].

## Conclusion

Our study demonstrates that the continuation of ASA in patients undergoing VP shunt surgery for iNPH, including those identified as high thromboembolic risk by preoperative cardiology evaluation, does not significantly increase the risk of postoperative hemorrhages. Importantly, we found no significant difference in hemorrhagic complications between patients who continued ASA and those who did not use it, suggesting that ASA discontinuation may not be necessary in high-risk patients. These findings support the safe perioperative use of ASA in this population. Further multicenter studies with larger cohorts and extended follow-up are essential to validate these results and provide a deeper understanding of both hemorrhagic and thromboembolic outcomes.

## Data Availability

No datasets were generated or analysed during the current study.

## References

[1] Ishida T, Murayama T, Kobayashi S (2023) Current research of idiopathic normal pressure hydrocephalus: Pathogenesis, diagnosis and treatment. World J Clin Cases 11(16):3706–3713. 10.12998/wjcc.v11.i16.3706 37383114 10.12998/wjcc.v11.i16.3706 PMC10294169

[2] Pedro MKF, da Sılva JFC, da Rocha SFB et al (2019) Salomón Hakim: the man behind normal pressure hydrocephalus. Arq Neuropsiquiatr. 77(10):746–748. 10.1590/0004-282x20190096 31664351 10.1590/0004-282X20190096

[3] Hakim S, Adams RD (1965) The special clinical problem of symptomatic hydrocephalus with normal cerebrospinal fluid pressure. J Neurol Sci 2(4):307–327. 10.1016/0022-510X(65)90016-X 5889177 10.1016/0022-510x(65)90016-x

[4] Courville E, Rumalla K, Kazim SF et al (2024) Risk Analysis Index as a preoperative frailty tool for elective ventriculoperitoneal shunt surgery for idiopathic normal pressure hydrocephalus. J Neurosurg 140(4):1110–1116. 10.3171/2023.7.JNS23767 38564806 10.3171/2023.7.JNS23767

[5] Sohail A, Bajwa MH, Virani QUA, Tariq A, Hussain N, Shamim SM (2024) Is endoscopic third ventriculostomy a viable treatment option for normal pressure hydrocephalus? A systematic review. Surg Neurol Int. 15:154. 10.25259/SNI_127_2024 38840608 10.25259/SNI_127_2024 PMC11152536

[6] Fiaschi P, Iaccarino C, Stefini R, Prior E, Prior A, Zona G (2021) Clinical practice for antiplatelet and anticoagulant therapy in neurosurgery: data from an Italian survey and summary of current recommendations – part I, antiplatelet therapy. Neurosurg Rev 44(1):485–493. 10.1007/s10143-019-01229-7 31953783 10.1007/s10143-019-01229-7

[7] Birkeland P, Lauritsen J, Poulsen FR (2015) Aspirin is associated with an increased risk of subdural hematoma in normal-pressure hydrocephalus patients following shunt implantation. J Neurosurg 123(2):423–426. 10.3171/2014.11.JNS14804 25555087 10.3171/2014.11.JNS14804

[8] Hanalioglu S, Sahin B, Sahin OS et al (2020) Effect of perioperative aspirin use on hemorrhagic complications in elective craniotomy for brain tumors: results of a single-center, retrospective cohort study. J Neurosurg 132(5):1529–1538. 10.3171/2018.12.JNS182483 30952120 10.3171/2018.12.JNS182483

[9] Rahman M, Donnangelo LL, Neal D, Mogali K, Decker M, Ahmed MM (2015) Effects of Perioperative Acetyl Salicylic Acid on Clinical Outcomes in Patients Undergoing Craniotomy for Brain Tumor. World Neurosurg 84(1):41–47. 10.1016/j.wneu.2015.02.016 25727304 10.1016/j.wneu.2015.02.016

[10] Lee AT, Gagnidze A, Pan SR et al (2017) Preoperative Low-Dose Aspirin Exposure and Outcomes After Emergency Neurosurgery for Traumatic Intracranial Hemorrhage in Elderly Patients. Anesth Analg 125(2):514–520. 10.1213/ANE.0000000000002053 28504994 10.1213/ANE.0000000000002053

[11] Rychen J, Saemann A, Fingerlin T et al (2022) Risks and benefits of continuation and discontinuation of aspirin in elective craniotomies: a systematic review and pooled-analysis. Acta Neurochir (Wien) 165(1):39–47. 10.1007/s00701-022-05416-2 36376767 10.1007/s00701-022-05416-2 PMC9840583

[12] Greuter L, Ullmann M, Mariani L, Guzman R, Soleman J (2019) Effect of preoperative antiplatelet or anticoagulation therapy on hemorrhagic complications in patients with traumatic brain injury undergoing craniotomy or craniectomy. Neurosurg Focus 47(5):E3. 10.3171/2019.8.FOCUS19546 31675713 10.3171/2019.8.FOCUS19546

[13] Rashidi A, Lilla N, Skalej M, Sandalcioglu IE, Luchtmann M (2021) Impact of acetylsalicylic acid in patients undergoing cerebral aneurysm surgery – should the neurosurgeon really worry about it? Neurosurg Rev 44(5):2889–2898. 10.1007/s10143-021-01476-7 33495921 10.1007/s10143-021-01476-7 PMC8490225

[14] Garcia Rodriguez LA, Cea-Soriano L, Martin-Merino E, Johansson S (2011) Discontinuation of low dose aspirin and risk of myocardial infarction: case-control study in UK primary care. BMJ. 343:d4094–d4094. 10.1136/bmj.d4094 21771831 10.1136/bmj.d4094 PMC3139911

[15] Sundström J, Hedberg J, Thuresson M, Aarskog P, Johannesen KM, Oldgren J (2017) Low-Dose Aspirin Discontinuation and Risk of Cardiovascular Events. Circulation 136(13):1183–1192. 10.1161/CIRCULATIONAHA.117.028321 28947478 10.1161/CIRCULATIONAHA.117.028321

[16] Biondi-Zoccai GGL, Lotrionte M, Agostoni P et al (2006) A systematic review and meta-analysis on the hazards of discontinuing or not adhering to aspirin among 50 279 patients at risk for coronary artery disease. Eur Heart J 27(22):2667–2674. 10.1093/eurheartj/ehl334 17053008 10.1093/eurheartj/ehl334

[17] Rychen J, Weiger VF, Halbeisen FS et al (2023) Perioperative continuation or ultra-early resumption of antithrombotics in elective neurosurgical cranial procedures. Neurosurg Focus 55(4):E6. 10.3171/2023.7.FOCUS23357 37778052 10.3171/2023.7.FOCUS23357

[18] Birkeland P, Lauritsen J, Poulsen FR (2016) Subdural haematoma complicating shunting for normal pressure hydrocephalus in the setting of concomitant antiplatelet medication – a report of 11 cases. Br J Neurosurg 30(5):567–570. 10.3109/02688697.2016.1173196 27100934 10.3109/02688697.2016.1173196

[19] Gasslander J, Sundström N, Eklund A, Koskinen LOD, Malm J (2021) Risk factors for developing subdural hematoma: a registry-based study in 1457 patients with shunted idiopathic normal pressure hydrocephalus. J Neurosurg 134(2):668–677. 10.3171/2019.10.JNS191223 31923893 10.3171/2019.10.JNS191223

[20] Hamouda AM, Pennington Z, Shafi M et al (2024) Ventriculoperitoneal Shunt Placement Safety in Idiopathic Normal Pressure Hydrocephalus: Anticoagulated Versus Non-Anticoagulated Patients. World Neurosurg 186:e622–e629. 10.1016/j.wneu.2024.04.018 38604534 10.1016/j.wneu.2024.04.018

[21] Feletti A, d’Avella D, Wikkelsø C et al (2019) Ventriculoperitoneal Shunt Complications in the European Idiopathic Normal Pressure Hydrocephalus Multicenter Study. Oper Neurosurg 17(1):97–102. 10.1093/ons/opy232 30169650 10.1093/ons/opy232

[22] Sundström N, Lagebrant M, Eklund A, Koskinen LOD, Malm J (2018) Subdural hematomas in 1846 patients with shunted idiopathic normal pressure hydrocephalus: treatment and long-term survival. J Neurosurg 129(3):797–804. 10.3171/2017.5.JNS17481 29076787 10.3171/2017.5.JNS17481

[23] Gao F, Ge S, Cui W et al (2024) Risk factors for hemorrhage in patients with long-term aspirin therapy undergoing emergency external ventricular drainage/intracranial pressure probe placement. Heliyon 10(5):e26854. 10.1016/j.heliyon.2024.e26854 38463769 10.1016/j.heliyon.2024.e26854 PMC10920161

[24] Cekic E, Pinar E, Pinar M, Dagcinar A (2023) Deep Learning-Assisted Segmentation and Classification of Brain Tumor Types on Magnetic Resonance and Surgical Microscope Images. *World Neurosurg*. Published online November 2023. 10.1016/j.wneu.2023.11.073 10.1016/j.wneu.2023.11.073 38030068

